# Comparison of the effects of contrast-enhanced ultrasound and conventional ultrasound-guided radiofrequency ablation on benign thyroid nodules

**DOI:** 10.12669/pjms.39.6.8250

**Published:** 2023

**Authors:** Shouxing Xu, Huiling He, Meijuan Jiang

**Affiliations:** 1 Shouxing Xu, Department of Ultrasound Medicine, Department of Ultrasound Medicine, the Fourth Affiliated Hospital of Zhejiang University School of Medicine, Yiwu 322000, Zhejiang Province, P.R. China; 2 Huiling He, Department of Ultrasound Medicine, the Fourth Affiliated Hospital of Zhejiang University School of Medicine, Yiwu 322000, Zhejiang Province, P.R. China; 3 Meijuan Jiang, Department of Ultrasound Medicine, the Fourth Affiliated Hospital of Zhejiang University School of Medicine, Yiwu 322000, Zhejiang Province, P.R. China

**Keywords:** Benign thyroid nodules, Contrast-enhanced ultrasound, Radiofrequency ablation

## Abstract

**Objective:**

To compare the effect of contrast-enhanced ultrasound (CEUS) and conventional ultrasound-guided radiofrequency ablation (RFA) on benign thyroid nodules (BTNs).

**Methods:**

In this retrospective observational study, the data of 72 patients with BTNs who received RFA treatment in The Fourth Affiliated Hospital of Zhejiang University School of Medicine from January 2020 to December 2021 were retrospectively reviewed and selected. The records showed that 34 patients received RFA under the guidance of conventional ultrasound (conventional ultrasound group) and 38 patients received RFA under the guidance of CEUS (CEUS group). The effect of treatment, complications and recurrence of the two groups were compared and analyzed.

**Results:**

There was a smaller volume of thyroid nodules in the two groups immediately post-operation. The incidence of complications was lower in the CEUS group (5.26%) compared to the conventional ultrasound group (23.53%) (P<0.05). The recurrence rate at 6-months (0.00% *vs* 11.76%) and 12- months (2.63% *vs* 20.59%) post-operation was lower in the CEUS group compared to the conventional ultrasound group (P<0.05).

**Conclusions:**

Compared with conventional ultrasound, CEUS-guided RFA is effective in treating BTNs, with smaller postoperative nodule volume, reduced occurrence of surgical complications, and reduced recurrence rate of thyroid nodules.

## INTRODUCTION

Thyroid nodules are one of the most common thyroid diseases, mainly nodular lesions caused by local abnormal growth of thyroid cells. Generally, they are proliferative, benign nodules, and 4.0-6.5% are malignant.^1^ Typically, small and asymptomatic benign thyroid nodules do not require treatment and can be observed with follow-up.^1^

At present, there are a variety of treatments for benign thyroid nodules (BTNs), including surgery, drug treatment and RFA. Although surgery can effectively remove the nodules, it is traumatic and can be complicated, while drug treatment may require extended medication and cause adverse reactions.^2^,^3^ Ultrasound-guided radiofrequency ablation (RFA) is a commonly used minimally invasive method for the treatment of BTNs. RFA uses high-frequency alternating wave current to cause vibration and friction of polar molecules and ions within the tissue, resulting in increased friction, elevating the local temperature to 40~50^0^C, and promoting cell dehydration. When the temperature rises to 60^0^C, the cells coagulate and necrosis occurs. When the temperature rises to 100 ^0^C, the tissues carbonize, form a reaction zone, and inhibit the formation of new blood vessels.^4^,^5^ Imaging guidance should be applied when implementing RFA in order to achieve precise treatment. In the past, conventional ultrasound was commonly used in clinical practice, however only the shape and boundary of thyroid nodules can be visualized, and the accuracy of ablation is not ideal.^6^,^7^ By injecting contrast agent into the tissue microcirculation, contrast-enhanced ultrasound (CEUS) can accurately display the blood flow status of the microvessels in the nodule lesion area, monitor the ablation range, and further improve the ablation effect and safety.^8^

In recent years, our hospital has begun to apply CEUS to the RFA treatment of BTNs, and achieved good results. There are studies comparing microwave ablation and RFA, but few comparing CEUS and conventional ultrasound-guided RFA.^9^,^10^ Therefore, a retrospective study was carried out on 72 patients treated from January 2020 to December 2021. Our objective was to compare the effect of CEUS and conventional ultrasound-guided RFA on BTNs.

## METHODS

In this retrospective observational study, a total of 72 patients (37 males and 35 females) with BTNs who received RFA treatment in the Fourth Affiliated Hospital of Zhejiang University School of Medicine from January 2020 to December 2021 were retrospectively selected by reviewing the clinical data. Their age ranged from 26 to 65 years, with an average of 47.53 ± 9.75 years. Based on the clinical data, there were 34 cases treated with RFA under the guidance of conventional ultrasound, and 38 cases treated with RFA under the guidance of CEUS.

### Inclusion criteria:

- Fine needle aspiration biopsy confirmed benign nodule by cytopathology.^11^
- Patients who meet the indications for RFA.^12^
- Number of nodules ≥2.
- Follow up more than 12 months after operation.
- No history of radiotherapy.

### Exclusion criteria:

- The thyroid nodule is close to the blood vessels, nerves, esophagus and other high-risk locations.
- Serious adhesion around the nodule.
- Patients with severe basic diseases, dysfunction of important organs (heart, liver, and kidney) or malignant tumors in these organs.
- Patients with severe risk of bleeding.
- Patients with contradiction to CEUS.
- Incomplete clinical data.

### Ethical Approval

The medical ethics committee of the Fourth Affiliated Hospital of Zhejiang University School of Medicine approved the study (approval number: K2023035; date: 2023-03-10).

### RFA treatment completed under the guidance of conventional ultrasound

The patient was asked to lie supine with their head back and neck fully exposed. The routine ultrasound examination was performed using an Esaote MyLabClassC ultrasound instrument (Esaote SpA, Genoa, Italy), with a probe frequency of 5~12MHz. After obtaining a stable two-dimensional image, the elastic mode was started and the area of interest was set. The patient was asked to hold their breath for three seconds, and the image was captured and stored.

The best puncture point was selected and the radiofrequency needle was inserted into the lesion under the guidance of ultrasound. The treatment was administered using STARmed radiofrequency ablation treatment system (Beijing Haiaosikang Technology Co., Ltd., Beijing, China). The needle was inserted into the deepest part of the nodule, from shallow to deep, and ablation was performed in multiple centers and planes. When the strong echo generated by thermal ablation completely covered the nodule, ablation was stopped.

### RFA performed under the guidance of CEUS

Preoperative preparation was the same as what was described previously in the conventional ultrasound examination. After the completion of conventional ultrasound examination, the ultrasound contrast mode was switched, and 2.4ml of SonoVue suspension (Bracco Imaging B.V., J20030117) was injected intravenously. When the enhancement of contrast agent was most obvious, the images were collected, and the meridians of nodules a, b, and c were measured. After the contrast agent receded, the puncture route was determined under the guidance of ultrasound.

The needle was inserted into the deepest part of the nodule, from shallow to deep, and ablation was performed in multiple centers and planes. When ultrasound showed that the high echo generated by ablation completely covered the nodule, ablation was stopped. Immediately, 15 minutes and 30 minutes after ablation, CEUS was performed again to observe the condition of the lesions in the patients of CEUS group. If there were residual lesions, supplementary ablation was needed.

The results of color Doppler ultrasound (Esaote MyLab 90, Genoa, Italy) were collected prior to, immediately post, three, six and twelve months post-operation. The reduction rate was calculated: nodule volume = (volume before ablation - volume at follow-up)/volume before ablation × 100%. Data concerning complications such as hematoma, hoarseness, parathyroid injury, and neck headache were collected. The recurrence was recorded at six and twelve months post-operation. Any echo and blood flow signals which were similar to the original nodule and were observed within the ablation area were considered recurrence.

### Statistical Analysis

SPSS V26.0 (SPSS Inc., Chicago, IL, USA) was used for statistical analysis. Categorical variables were presented as cases (n%), and were compared via Fisher’s exact test or Chi-squared tests. Continuous variables were presented as means±SD (standard deviation), and were compared via Student’s t-tests for normally distributed data or Mann-Whitney’s U tests for unnormally distributed data. Significance was set at *p*<0.05.

## RESULTS

A total of 72 patients were included in this study, all of whom received RFA treatment. RFA was performed in 34 cases under the guidance of conventional ultrasound, including 15 males and 19 females. Their ages ranged from 28 to 63 years, with an average of 48.79±9.22 years. The nodular volume was 3.5-6.1 ml with an average of 4.76±0.72 ml. There were 15 cases of the left nodule and 19 cases of the right nodule. RFA was performed under the guidance of CEUS in 38 cases, including 22 males and 16 females. Their ages ranged from 26 to 65 years, with an average of 46.39±10.18 years. The nodular volume was 3.5-6.2ml, with an average of 4.91 ± 0.81ml. There were 17 cases of the left nodule and 21 cases of the right nodule. There was no statistically significant difference in baseline data between the two groups (*P*>0.05; Table-I). Thyroid nodule volume immediately post-operation in both groups was smaller than that pre-operation (*P*<0.05), and the CEUS group was smaller than that in the conventional ultrasound group (*P*<0.05). The reduction rate of the CEUS group at three, six and twelve months post-operation was significantly higher than that of the conventional ultrasound group (P<0.05; Table-II). The incidence of complications within the CEUS group was 5.26%, which was lower than 23.53% in the conventional ultrasound group (*P*<0.05; Table-III). The recurrence rate in the CEUS group was lower than that in the conventional ultrasound group at 6 months (0.00% *vs* 11.76%) and 12 months (2.63% *vs* 20.59%) post-operation (*P*<0.05; Table-IV).

**Table-I:** Baseline data of the conventional ultrasound and CEUS groups.

Group	n	Gender (Male/Female)	Age (year)	Nodule location (n)	

				left	right
Conventional ultrasound group	34	15/19	48.79±9.22	13	21
CEUS group	38	22/16	46.39±10.18	19	19
*χ^2^*/*t*	-	1.6363	1.043	1.006	
*P*	-	0.243	0.300	0.316	

**Table-II:** Thyroid nodule volume change and volume reduction rate between the two groups.

Group	Preoperative nodule volume (ml)	Nodular volume immediately after operation (ml)	Reduction rate of nodule volume 3 months after operation (%)	Reduction rate of nodule volume 6 months after operation (%)	Reduction rate of nodule volume 12 months after operation (%)
Conventional ultrasound group (n=34)	4.76±0.72	2.17±0.58^a^	45.23±5.71	75.41±6.33	83.70±7.02
CEUS group(n=38)	4.91±0.81	1.84±0.70^a^	52.21±6.02	79.79±6.62	88.79±7.19
*t*	-0.819	2.173	-5.025	-2.858	-3.028
*P*	0.416	0.033	<0.000	0.006	0.003

***Note:*** Compared with the group before operation,

a P<0.05.

**Table-III:** Complications between the conventional ultrasound and CEUS groups [n (%)].

Group	n	Complication				Total

		hematoma	hoarseness	Parathyroid injury	Neck and head pain	
Conventional ultrasound group	34	3 (8.82)	1 (2.94)	2 (5.88)	2 (5.88)	8 (23.53)
CEUS group	38	1 (2.63)	0 (0.00)	1 (2.63)	0 (0.00)	2 (5.26)
*χ^2^*	-	-	-	-	-	5.006
*P*	-	-	-	-	-	0.025

**Table-IV:** Recurrence between the conventional ultrasound and CEUS groups [n (%)].

Group	n	Six months after operation	12 months after operation
Conventional ultrasound group	34	4 (11.76)	7 (20.59)
CEUS group	38	0 (0.00)	1 (2.63)
*χ^2^*	-	4.734	5.858
*P*	-	0.030	0.016

## DISCUSSION

Our study showed that the use of CEUS technology to assist RFA in the ablation of benign thyroid nodules can further reduce the complication and recurrence rate compared to conventional ultrasound. Although thyroid lobectomy is the preferred treatment for patients with BTNs, in clinical practice, surgical resection can leave permanent scars and damage to thyroid function, possibly resulting in the need for long-term medication.^13^ In recent years, with the continuous development and progress of minimally invasive treatment technology, RFA has gradually been used in the treatment of patients with BTNs, which may eliminate compression symptoms, avoid surgery and has few complications.^6^

Moreover, with the development and progress of imaging technology, the application scope of CEUS technology in RFA continues to expand, further improving the accuracy of ablation treatment.^14^ Jin H et al^15^ compared and analyzed the effects of traditional surgery and ultrasound-guided RFA in the treatment of benign thyroid nodules, and found that there was no significant difference in the complications between the two. However, thyroid thermal ablation was superior to traditional thyroidectomy in terms of patient satisfaction, postoperative quality of life and hospital stay. In addition, Schiafino S et al^16^ evaluated the reproducibility of RFA volume of benign thyroid nodules, CEUS had a higher reproducibility in evaluating the RFA volume of benign thyroid nodule. CEUS is a kind of vascular enhancement technology and after injecting the contrast agent into the circulatory system, the contrast agent can produce resonance in the sound field, clearly display the blood flow signal, and increase the image.

When it is applied to the RFA treatment of thyroid nodules, it can accurately display the range of nodules, and can be used to evaluate the effectiveness of the ablation, effectively ablate the small nodules, improve the thoroughness of treatment, and reduce the recurrence rate of nodules.^17^,^18^ The recurrence rate in our findings is basically consistent with the findings by Zhu et al^19^ and Zhang et al^20^. Not only that, Deandrea M et al^21^ showed that in RFA of thyroid nodules, the vaporization produced by ablation can interfere with conventional ultrasound, and conventional ultrasound is difficult to accurately detect microvessels, while CEUS can make up for the deficiency of conventional ultrasound.

Although RFA is a kind of minimally invasive surgery, puncture, ablation and other operations can still result in trauma, causing complications such as hematoma and hoarseness, affecting overall surgical rehabilitation.^22^ In this study, the incidence of complications in the CEUS group is lower than that in the conventional ultrasound group, which is consistent with the results by Jin Z et al.^23^ Both studies suggest that the application of CEUS can effectively reduce the incidence of complications in the ablation of patients with BTNs.

It’s possible that CEUS allows for an increased visualization of the thyroid nodule which improves the accuracy of the ablation and further improves the safety of ablation treatment, and reduces the occurrence of complications.^24^,^25^ Based on the above analysis, when treating BTNs clinically, we suggest that those who meet the following conditions can actively choose CEUS-guided RFA to further improve the clinical benefits of patients: Patients whose nodules are benign, who have no prior history of radiation therapy, who have autonomic functional nodules, hyperthyroidism symptoms, or conscious symptoms. At the same time, attention should be paid to the elimination of contraindications of RFA treatment which are as below: Patients with huge retrosternal goiter, or nodules located retrosternal, vocal cord insufficiency contralateral to the lesion, coagulation impairment, or organ insufficiency.

### Limitations

This study was a single-center retrospective analysis, with a small sample size. Additionally, the follow-up time was short with a limited number of observation indicators, which could make the conclusion subjective and one-sided. Furthermore, intraoperative blood loss is associated with length of hospital stay, complication rate, and patient survival. Therefore, management of bleeding associated with RFA of BTNs is an important aspect, but it was not investigated in this study.

## CONCLUSION

Compared with conventional ultrasound, CEUS-guided RFA is effective in treating BTNs, with smaller postoperative nodule volume, reduced occurrence of surgical complications, and reduced recurrence rate of thyroid nodules.

### Authors’ contributions:

**SX:** Conceived and designed the study.

**HH** and **MJ:** Collected the data and performed the analysis.

**SX:** Was involved in the writing of the manuscript and is responsible for the integrity of the study.

All authors have read and approved the final manuscript.

## References

[1] Tamhane S, Gharib H (2016). Thyroid nodule update on diagnosis and management. Clin Diabetes Endocrinol.

[2] Ma T, Zhang S, Huang D, Zhang G, Chen B, Zhang N (2022). Endoscopicassisted lateral neck dissection and open lateral neck dissection in the treatment of lateral neck lymph node metastasis in papillary thyroid carcinoma:A comparison of therapeutic effect. Pak J Med Sci.

[3] Mahno NE, Wahab NA, Md Latar NH (2022). The role of intra-operative parathyroid hormone assay in non-localized adenoma. Pak J Med Sci.

[4] Hartley-Blossom Z, Alam M, Stone J, Iannuccilli J (2020). Microwave Ablation in the Liver:An Update. Surg Technol Int.

[5] Guo DM, Chen Z, Zhai YX, Su HH (2021). Comparison of radiofrequency ablation and microwave ablation for benign thyroid nodules:A systematic review and meta-analysis. Clin Endocrinol (Oxf).

[6] Ram N, Hafeez S, Qamar S, Hussain SZ, Asghar A, Anwar Z (2015). Diagnostic validity of ultrasonography in thyroid nodules. J Pak Med Assoc.

[7] Zhang H, Wang J, Guo R (2020). Application Value of Color Doppler Ultrasound and Ultrasound Contrast in the Differential Diagnosis of Ovarian tumor. Pak J Med Sci.

[8] Blackburn J, Weaver L, Cohen L, Menachemi N, Rusyniak DE, Unroe KT (2021). Community Coronavirus Disease 2019 Activity Level and Nursing Home Staff Testing for Active Severe Acute Respiratory Syndrome Coronavirus 2 Infection in Indiana. J Am Med Dir Assoc.

[9] Cao J, Fan P, Wang F, Shi S, Liu L, Yan Z (2022). Application of contrast-enhanced ultrasound in minimally invasive ablation of benign thyroid nodules. J Interv Med.

[10] Jin H, Fan J, Lu L, Cui M (2021). A Propensity Score Matching Study Between Microwave Ablation and Radiofrequency Ablation in Terms of Safety and Efficacy for Benign Thyroid Nodules Treatment. Front Endocrinol (Lausanne).

[11] Paschke R, Cantara S, Crescenzi A, Jarzab B, Musholt TJ, Sobrinho Simoes M (2017). European Thyroid Association Guidelines regarding Thyroid Nodule Molecular Fine-Needle Aspiration Cytology Diagnostics. Eur Thyroid J.

[12] Lee M, Baek JH, Suh CH, Chung SR, Choi YJ, Lee JH (2021). Clinical practice guidelines for radiofrequency ablation of benign thyroid nodules:a systematic review. Ultrasonography.

[13] Gibelin H, Sierra M, Mothes D, Ingrand P, Levillain P, Jones C (2004). Risk factors for recurrent nodular goiter after thyroidectomy for benign disease:case-control study of 244 patients. World J Surg.

[14] Bernardi S, Giudici F, Colombin G, Cavallaro M, Stacul F, Fabris B (2021). Residual vital ratio predicts 5-year volume reduction and retreatment after radiofrequency ablation of benign thyroid nodules but not regrowth. Int J Hyperthermia.

[15] Jin H, Lin W, Lu L, Cui M (2021). Conventional thyroidectomy vs thyroid thermal ablation on postoperative quality of life and satisfaction for patients with benign thyroid nodules. Eur J Endocrinol.

[16] Schiaffino S, Serpi F, Rossi D, Ferrara V, Buonomenna C, Alì M (2020). Reproducibility of Ablated Volume Measurement Is Higher with Contrast-Enhanced Ultrasound than with B-Mode Ultrasound after Benign Thyroid Nodule Radiofrequency Ablation-A Preliminary Study. J Clin Med.

[17] Fang F, Gong Y, Liao L, Ye F, Zuo Z, Qi Z (2021). Value of Contrast-Enhanced Ultrasound in Partially Cystic Papillary Thyroid Carcinomas. Front Endocrinol (Lausanne).

[18] Zhao W, Lu R, Yin L, Guo R (2021). The value of superb microvascular imaging (SMI) scoring assignment method in differentiating benign and malignant thyroid nodules by conventional ultrasound. Clin Hemorheol Microcirc.

[19] Zhu Y, Jiao Z, Zhu L, Xie F, Song Q, Yan L (2022). A New Perspective for Predicting the Therapeutic Success of RFA in Solid BTNs:Quantitative Initial RFA Ratio by Contrast-Enhanced Ultrasound. Front Endocrinol (Lausanne).

[20] Zhang M, Luo Y, Zhang Y, Tang J (2016). Efficacy and Safety of Ultrasound-Guided Radiofrequency Ablation for Treating Low-Risk Papillary Thyroid Microcarcinoma:A Prospective Study. Thyroid.

[21] Deandrea M, Sung JY, Limone P, Mormile A, Garino F, Ragazzoni F (2015). Efficacy and Safety of Radiofrequency Ablation Versus Observation for Nonfunctioning Benign Thyroid Nodules:A Randomized Controlled International Collaborative Trial. Thyroid.

[22] Daniels SP, Xu HS, Hanna A, Greenberg JA, Lee KS (2021). Ultrasoundguided microwave ablation in the treatment of inguinal neuralgia. Skeletal Radiol.

[23] Jin Z, Zhu Y, Xie F, Zhang Y, Li N, Luo Y (2021). Contrast agent retention features in contrast-enhanced ultrasound:diagnostic performance for the prediction of papillary thyroid carcinoma. Clin Imaging.

[24] Karatay E, Javadov M (2021). The role of ultrasound measurements and cosmetic scoring in evaluating the effectiveness of ethanol ablation in cystic thyroid nodules. Int J Clin Pract.

[25] Liu Q, Cheng J, Li J, Liu L, Li H (2021). Clinical Study of Virtual Reality Augmented Technology Combined with Contrast-Enhanced Ultrasound in the Assessment of Thyroid Cancer. J Healthc Eng.

